# Mutant selection window of clarithromycin for clinical isolates of *Helicobacter pylori*

**DOI:** 10.1186/s12866-019-1558-8

**Published:** 2019-08-05

**Authors:** Zi-Han Feng, Ling Fan, Jing Yang, Xing-Yue Huo, Yan Guo, Yi Zhang, Chun-Hui Lan

**Affiliations:** 1 College of Basic Medical Sciences, Army Medical University, Chongqing, 400042 China; 2 Department of Gastroenterology, Daping Hospital, Army Medical University, 10 Changjiang Branch Road, Chongqing, 400042 China

**Keywords:** *Helicobacter pylori*, Clarithromycin, MPC, MSW, Antibiotic resistance

## Abstract

**Background:**

Clarithromycin-resistance is becoming a global health concern in the treatment of *Helicobacter pylori* (*H. pylori*). The mutant prevention concentration (MPC) represent the propensities of antimicrobial agents to select resistant mutants. The concentration range between the minimum inhibitory concentration (MIC) and the MPC is defined as mutant selection window (MSW). In this study, we aimed to determine the cause of increasing clarithromycin resistance by investigating the MSW for clinical isolates of *H. pylori*.

**Results:**

A retrospective subgroup, which included 68 clarithromycin-sensitive *H. pylori* strains, was selected from a double-blind trial. The MICs and MPCs were determined using agar plate assays. Genotypic tests were performed using Sanger sequencing. All isolates were wild-type, and 33.82% (23/68) had a 0.016 mg/L MIC, 45.59% (31/68) had a 0.031 mg/L MIC, 16.18% (11/68) had a 0.062 ≤ MIC ≤ 0.125 mg/L, and 4.41% (3/68) had a 0.25 mg/L MIC. The MPC_50/90_ (mg/L) of the isolates were: 0.062/0.125, 0.125/0.5, 0.25/0.25 and 1/2, respectively. The MPCs showed a moderate correlation with the MICs (*r*_*s*_ = 0.65, *P* < 0.0001). Using published data and MPC_90_, we calculated the time inside the MSW (T_MSW_) for low- and high-dose (200 or 500 mg bid) clarithromycin that were 6 and 0 h, 24 and 4 h, 15 and 2 h, 5 and 17 h for the strains with MICs (mg/L) of 0.016, 0.031, 0.062–0.125, and 0.25, respectively.

**Conclusions:**

This study showed that in the clarithromycin-sensitive clinical isolates of *H. pylori*, low-dose clarithromycin may lead to decreased drug sensitivity or even clarithromycin resistance; strains with a 0.25 mg/L MIC display a high risk of treatment failure.

## Background

Clarithromycin is the most powerful antimicrobial agent used in the treatment of *Helicobacter pylori* (*H. pylori)* infection [1], yet it is recognized as a major cause of peptic ulcers [2]. Treatment of *H. pylori* with clarithromycin alone is far from satisfactory [3], while the performance of clarithromycin is markedly improved when combined with other agents, particularly acid suppressive agents [4]. Several clarithromycin-based triple therapies including clarithromycin, amoxicillin, and ranitidine were previously found to be highly effective [5, 6]. Now, clarithromycin-containing bismuth quadruple therapies are recommended by many international guidelines in the treatment of *H. pylori* infection [7–11]. Therefore, clarithromycin continues to play an important role in *H. pylori* treatment.

Recently, clarithromycin resistance has been steadily increasing worldwide. In addition, resistance to clarithromycin has been ranked as a high priority pathogen by the World Health Organization [12]. The eradication rate has been severely reduced with clarithromycin-containing bismuth quadruple therapies [7]. While it is known that a history of prior clarithromycin use can influence the failure rate of standard triple therapies in patients [13], the mechanisms by which it affects the MICs and the reasoning for the skyrocketing increase in resistance to clarithromycin is unknown [1].

The mutant prevention concentration (MPC) represents the lowest drug concentration that prevents first-step mutant subpopulation growth in a large bacterial population, usually more than 10^10^ colony forming unit (CFU)/mL bacteria [14]. The mutant selection window (MSW) is the range of antibiotic concentrations above the minimum inhibitory concentration (MIC) and below the MPC. If the maximum serum concentration is within the MSW, the growth of susceptible bacteria is suppressed, and the non-susceptible mutant variants are selectively enriched, resulting in eradication failure [15]. The MPC theory has been used to compare drug susceptibility and to investigate the relationships between pharmacokinetic and pharmacodynamic (PKPD) values and resistance in various of bacteria, such as *Mannheimia haemolytica* [16], *Escherichia coli* [17], *Staphylococcus aureus* [18], *Salmonella enterica* [19], and *Pseudomonas aeruginosa* [20]. As of now, there are no published reports on the MPC of clarithromycin for clinical isolates of *H. pylori*.

In this study, we aim to investigate the MPC of clarithromycin-sensitive clinical isolates of *H. pylori*. To investigate the reasoning for the increased rates of clarithromycin resistance, the MPC was combined with the PKPD values for low- and high-dose (200 or 500 mg bid) clarithromycin to calculate changes in the MICs during the treatment of respiratory and *H. pylori* infections.

## Results

Clinical characteristics of patients are provided in Table 1. Genotypic tests showed that all isolates were wild-type. 33.82% (23/68) had a 0.016 mg/L MIC, 45.59% (31/68) had a 0.031 mg/L MIC, 16.18% (11/68) had a 0.062 ≤ MIC≤0.125 mg/L, 4.41% (3/68) had a 0.25 mg/L MIC. There were no significant differences between the eradication rate of strains with different MICs.

**Table 1:** Baseline demographics and clinical and characteristics of patients with different MIC values

	MIC distribution data				Total	*P* -value
Characteristics	≤0.016	0.031	0.062–0.125	0.25		
Gender (male/female)	11/12	14/17	6/5	2/1	33/35	0.9185
Age, mean ± SD (years)	45 ± 11.43	41 ± 10.84	42± 10.80	38 ± 12.70	42 ± 11.06	0.5475
Cigarette smoking (yes/no)	6/17	8/23	2/9	2/1	18/50	0.4952
Alcohol drinking (yes/no)	9/14	13/18	6/5	0/3	28/40	0.4737
Endoscopic findings (PUD/NUD)	4/19	8/23	5/6	0/3	17/51	0.2890
Eradication rate	100% (23/23)	93.55% (29/31)	90.91% (10/11)	100% (3/3)	95.59% (65/68)	0.4736
BQT	100% (17/17)	91.67% (22/24)	87.5% (7/8)	100% (3/3)	94.23% (49/52)	0.4923
Others	100% (6/6)	100% (7/7)	100% (3/3)	None	100% (16/16)	1.0000

*SD* standard deviation, *PUD* peptic ulcer disease, *NUD* non-ulcer dyspepsia, *BQT* clarithromycin-containing bismuth quadruple therapy. Concentrations are in mg/L

The MPC_50_ values for clarithromycin in the strains with different MICs were 0.062, 0.125, 0.25 and 1 mg/L, respectively. The MPC_90_ values were 0.125, 0.5, 0.25 and 2 mg/L, respectively (Table 2). The MPCs showed a moderate correlation with the MICs (*r*_*s*_ = 0.65, *P* < 0.0001). The MIC_90_ and MPC_90_ represented the boundaries of the MSW [21]. The window sizes were 16 (0.016, 0.125), 16 (0.031, 0.5), 4(0.062, 0.25), and 8(0.25, 2.0) for the strains with the MICs (mg/L) of 0.016, 0.031, 0.062–0.125, and 0.25, respectively. Strains with smaller MICs had bigger MSW boundaries.

**Table 2:** MIC/MPC distribution for clarithromycin with clinical isolates of *H. pylori* (*n* = 68)

	MPC distribution data							
MICs	0.062	0.125	0.25	0.5	1	2	MPC_50_	MPC_50_
0.016	17	5	1				0.062	0.125
0.031		9	17	5			0.125	0.5
0.062–0.125		5	5	1			0.25	0.25
0.25					2	1	1	2
Total	17	14	23	10	3	1	0.25	0.5

*MIC* minimum inhibitory concentration, *MPC* mutant prevention concentrations, *MIC*_*50*_ drug concentration at which 50% of strains are inhibited, *MIC*_*90*_ drug concentration at which 90% of strains are inhibited, *MPC*_*50*_ drug concentration at which 50% of strains are inhibited, *MPC*_*90*_ drug concentration at which 90% of strains are inhibited. Concentrations are in mg/L

The pharmacodynamic indices were determined by combining the MPC and MIC values with the pharmacokinetic parameters, such as area under curve over 24 h (AUC_24_) and serum maximum concentration (C_max_), as shown in Table 3. The C_max_/MPC_90,_ AUC_24_ /MPC_90_ and time inside the MSW (T_MSW_) were calculated for two dosages in the treatment of *H. pylori* infection (500 mg bid), and respiratory infections (200 mg bid).

**Table 3:** Pharmacokinetic and pharmacodynamic (PKPD) values for clarithromycin

	Dosage 2 × 200 mg C_max_^a^ = 0.36 AUC_24_ = 4.20						
MICs	C_max_/MIC_90_	C_max_/MPC_90_	AUC_24_/MIC_90_	AUC_24_/MPC_90_	T > MIC_90_	T > MPC_90_	T_MSW_
0.016	22.50	2.88	262.50	33.60	24	~ 18	~ 6
0.031	11.61	0.72	135.48	8.40	24	0	24
0.062–0.125	5.81	1.44	67.74	16.80	~ 20	~ 5	~ 15
0.25	1.44	0.18	16.80	2.10	~ 5	0	~ 5
	Dosage 2 × 500 mg C_max_^b^ = 2.85 AUC_24_ = 41.72						
	C_max_/MIC_90_	C_max_/MPC_90_	AUC_24_/MIC_90_	AUC_24_/MPC_90_	T > MIC_90_	T > MPC_90_	T_MSW_
0.016	178.13	22.80	2607.50	333.76	24	24	0
0.031	91.94	5.70	1345.81	83.44	24	~ 20	~ 4
0.062–0.125	45.97	11.40	672.90	166.88	24	~ 22	~ 2
0.25	11.40	1.43	166.88	20.86	~ 22	~ 5	~ 17

*C*_*max*_ serum maximum concentration, *AUC*_*24*_ area under curve over a 24 h time period, *T*_*MSW*_ time inside the mutant selection window (h). Concentrations are in mg/L

^a^C_max_ was determined using a high-performance liquid chromatographic procedure and did not include 4-hydroxy-clarithromycin

^b^C_max_ was determined using liquid chromatography tandem mass spectrometry and did not include 4-hydroxy-clarithromycin

When the dosage of clarithromycin is 200 mg bid, the C_max_/MPC_90_ is 2.88 for strains with the MICs of 0.016 mg/L; 0.72 for strains with the MICs of 0.031 mg/L; 1.44 for strains with the MICs ranging from 0.062 to 0.125 mg/L; and 0.18 for strains with the MICs of 0.25 mg/L. When the dosage of clarithromycin is 500 mg bid, the C_max_/MPC_90_ is 22.80 for strains with the MICs of 0.016 mg/L; 5.70 for strains with the MICs of 0.031 mg/L; 11.40 for strains with the MICs ranging from 0.062 to 0.125 mg/L; and 1.43 for strains with the MICs of 0.25 mg/L.

When the dosage of clarithromycin is 200 mg bid, the AUC_24_/MPC is 33.60 for strains with the MICs of 0.016 mg/L; 8.4 for strains with the MICs of 0.031 mg/L; 16.80 for strains with the MICs ranging from 0.062 to 0.125 mg/L; 2.10 for strains with the MICs of 0.25 mg/L. When the dosage of clarithromycin is 500 mg bid, the AUC_24_/MPC is 333.76 for strains with the MICs of 0.016 mg/L; 83.44 for strains with the MICs of 0.031 mg/L;166.88 for strains with the MICs ranging from 0.062 to 0.125 mg/L; 20.86 for strains with the MICs of 0.25 mg/L.

When the dosage of clarithromycin is 200 mg bid, the T_MSW_ (h) is 6 for strains with the MICs of 0.016 mg/L; 24 for strains with the MICs of 0.031 mg/L;15 for strains with the MICs ranging from 0.062 to 0.125 mg/L; and 5 for strains with the MICs of 0.25 mg/L. The T_MSW_ values indicate that non-susceptible mutant subpopulations are more likely to be selectively enriched with the low-dose clarithromycin. As a result, the MICs increase with low-dose clarithromycin. For the strains with the MICs of 0.25 mg/L and MPC_90_ of 2 mg/L, the MICs might increase to 2 mg/L, since the subpopulations were able to survive in the concretions of 1 mg/L.

When the dosage of clarithromycin is 500 mg bid, the T_MSW_ (h) is 0 for strains with the MICs of 0.016 mg/L; 4 for strains with the MICs of 0.031 mg/L; 2 for strains with the MICs ranging from 0.062 to 0.125 mg/L; and 17 for strains with the MICs of 0.25 mg/L. The T_MSW_ values indicate that non-susceptible mutant subpopulations are less likely to be selectively enriched with the high-dose clarithromycin. However, for the strains with the MICs of 0.25 mg/L, the T_MSW_ is much higher, presenting a high risk for treatment failure.

## Discussion

*H. pylori*, a major causative agent of chronic gastritis and peptic ulcers, is related to the pathogenesis of gastric mucosa-associated lymphoid tissue lymphomas and gastric adenocarcinomas [22, 23]. Stomach cancer is the second leading cause of cancer-related death in East Asia with China accounting for nearly 50% of all new stomach cancer cases diagnosed worldwide each year [6]. It is believed that eradication of *H. pylori* may prevent some of the complications associated with ulcer and gastric cancers [24]. Classical clarithromycin-containing triple therapy has been recognized as the first-line treatment for *H. pylori* infection, yet the effectiveness of this triple therapy has dropped from 88.54 to 71.13% in recent years. This decline in effectiveness has been attributed to the development of clarithromycin resistance in some *H. pylori* strains [7]. *H. pylori* resistance against clarithromycin is primarily attributed to point mutations in the variable region of the *23S rRNA* gene (V region). The mutations result in a conformational change in the clarithromycin binding sites, resulting in lower binding and decreased drug sensitivity [25, 26]. Strains are considered to be resistant to clarithromycin when the MIC becomes ≥1 mg/L [27]. In this study, we discovered that the MICs increase with low-dose clarithromycin. For the strains with the MICs of 0.25 mg/L and MPC_90_ of 2 mg/L, the non-susceptible mutant subpopulations were resistant to clarithromycin.

Two conditions are required for the phenotypic development of clarithromycin resistance in bacteria. The first condition is the emergence of drug-resistant mutant strains. Secondly, the drug-resistant mutant strains must be selectively enriched [14]. For *H. pylori,* the frequency of *23S rRNA* gene mutation is about 3 × 10^− 9^ and the bacterial load of *H. pylori* is between 10^8^ and 10^11^ CFU in the stomach [28]. For this reason, clarithromycin-resistant mutants exist in almost all *H. pylori*-infected patients. The clarithromycin resistance mutants are not normally detected by standard susceptibility testing because it requires an inoculum size of 10^6^ CFU [27]. Unlike many other bacteria, *H. pylori* cannot be destroyed by the immune system, so that the drug-resistant mutant strains can be selectively enriched [2, 29]. Previously, the frequency of clarithromycin resistance mutations was found to be 3 × 10^− 9^ for the UA802 strain, the MIC of which was 0.02 mg/L [18]. In the current study, the frequency for clarithromycin-sensitive *H. pylori* to become resistant in one step was less than 10^− 10^ for 65/68 (95.59%) wild-type clinical isolates of *H. pylori*. Indeed, the MICs increased steadily. Before the MPC theory was proposed, researchers realized that the combination of two antibiotics was needed to achieve optimal cure rate [5, 6]. Two drugs with separate bacterial targets can close the MSW if the pharmacokinetic parameters are optimized [15]. However, co-administration of clarithromycin with other drugs can only reduce the time in the MSW but not close it, since the pharmacokinetic parameters are not usually optimized. If scientists can optimize the dosage of clarithromycin to close the MSW, the eradication rate will likely increase and making it possible to avoid secondary resistance with less dosage.

We found that when clarithromycin was used in the treatment of respiratory infections, for the strains with the MICs of 0.031 mg/L and the MPC_90_ of 0.5 mg/L, the clarithromycin concentrations were dropped in the MSW during the entire dosing interval. In other words, the MSW was opened the entire time. The non-susceptible mutant subpopulations were able to survive in the concertation of 0.25 mg/L. When they were enriched to 10^10^ CFU, the new variants might present and be enriched again. For the strains with the MICs of 0.25 mg/L and the MPC_90_ of 2 mg/L, the T_MSW_ was only 5 h, while clarithromycin concentrations were under the MPC_90_ the entire time. It may need more time to enrich the variants, which can survive in the concertation of 1 mg/L. However, these will become clarithromycin-resistant strains. Before that, there might have been a mixture of stains with different MICs values.

When clarithromycin was used in the treatment of *H. pylori* infections, for most strains, the clarithromycin concentrations were above the MSW during the entire dosing interval. In other words, the MSW was closed the entire time. For the strains with the MICs of 0.25 mg/L and the MPC_90_ of 2 mg/L, the resistant mutants can easily be enriched in theory. While there were no significant differences between the eradication rate of them and that of other strains in this study. This may be due to the use of bismuth and amoxicillin, which may affect the enrichment of resistant mutants. Also, the number of the patients may have been too small to find the difference.

A history of prior clarithromycin use was considered to influence the failure rate of standard triple therapy in patients [13]. In this study, we found that the non-susceptible mutant variants might be enriched, resulting in a decrease in drug sensitivity. Despite most MPC values of the wild-type strains were under the MIC breakpoint of 1 mg/L, the frequent low-dosage of the clarithromycin might steadily increase the MICs. When it reaches 0.25 mg/L, the variants of which will be able to survive in the concertation of 1 mg/L. We can say that the MICs increase in every exposure to clarithromycin, unless all variants are killed. In return, it may be helpful to decrease the number of clarithromycin exposures and to avoid low-dosages when possible. If patients have used low-dosage clarithromycin several times in the past, clarithromycin should not be considered as the choice of the *H. pylori* treatment.

Despite MPC has many advantages over the MIC in theory, there are three limitations that may affect the clinical applications in the treatment of *H. pylori* infection. Firstly, the results of MPC and genotypic testing cannot reflect the actual characteristics of *H. pylori* in the whole stomach, even if the inoculum size reaches the bacterial load. For one thing, infection with both wild-type strains and variants of *H. pylori* strains is common during long-time chronic infection [30]. It is important to note that the MPC test theory is imperfect and has some level of inaccuracy. For example, the variants of *H. pylori* strains may have been lost in the recovery and subculture process, causing phenotypic susceptible strains revealed resistant genotype or genotypic resistant strains revealed susceptible phenotype, indicating that the result may not reflect all the variants of *H. pylori* strains in the stomach. Similar findings have been reported in the biopsies obtained from children and fecal specimens from young adults [31–33]. Secondly, the transformation of the *23S rRNA* gene may reduce the relevance of MPC values to clarithromycin resistance [34], since the spread of a clonal strain will reduce the importance of the emergence of new drug resistant strains [35]. Thirdly, the high bacteria load, the gastric pH level, the mucous layer, and the *H. pylori* intracellular life and colonization in niches with low antibiotic penetration can also affect the effectiveness of clarithromycin [36]. Additional in vivo and in vitro studies are needed to assess the application MSW theory in the treatment of *H. pylori* infection and the relationship between AUC_24_/MPC values and clarithromycin resistance.

## Conclusions

In summary, this is the first study investigating the MSW of clarithromycin for clinical isolates of phenotypically-sensitive *H. pylori*. Low-dose clarithromycin may lead to decreased drug sensitivity or even clarithromycin resistance. Strains with a 0.25 mg/L MIC display a high risk of treatment failure. Additional in vivo and in vitro studies are needed to confirm the relationship between MPC values and clarithromycin resistance.

## Methods

### Ethics statement

The study was approved by Ethics Committee of Daping Hospital, Army Medical University, Chongqing, China (Ethics Approval Number: 20, 2017). All participants provided written informed consent prior to the study.

### Bacterial strains and antimicrobial agents

A total of 68 clarithromycin-sensitive *H. pylori* clinical strains were used in this study. Two biopsy specimens from the antrum and the body were obtained during the endoscopic examination. They were mixed and then divided into two parts. One part was used for DNA extraction, while the other part was streaked onto an agar plate with selective medium containing polymyxin B, trimethoprim vancomycin, and nalidixic acid. All isolates were collected from Daping Hospital (Chongqing, China) and were susceptible to clarithromycin according to the recommended breakpoint of the Clinical & Laboratory Standards Institute (CLSI) [9]. *H. pylori* isolates were placed in cryopreservation media and stored at − 70 °C. Mueller–Hinton II agar medium (Becton Dickinson, Sparks, MD, USA) was supplemented with 5% defibrinated sheep blood without antibiotics. Clarithromycin (BioMerieux, St. Louis, MO, USA) was prepared according to the manufacturer’s recommendations.

### Genotypic test

Detection of clarithromycin resistance-associated mutations in the *23S rRNA* was conducted by polymerase chain reaction (PCR) and Sanger sequencing using *H. pylori 23S rRNA*-specific primers as reported by Noguchi et al [37]. The sequence of the purified product was compared with the arrangement of the clarithromycin-sensitive *H. pylori* strain ACTC43504 using a Sequence Scanner (Thermo Fisher, Waltham, MA, USA).

### MIC determination

The determination of MIC was performed according to CLSI guidelines and interpretations [27]. Isolates stored at − 70 °C were thawed and subcultured using Mueller-Hinton (MH) II agar medium supplemented with 5% defibrinated sheep blood. Isolated colonies were suspended in phosphate-buffered saline (PBS) and adjusted to a concentration of 1 × 10^8^ CFU/mL. Next, 10 μL of the bacterial suspension containing 1 × 10^6^ CFU of bacteria was added to each MH agar plates containing 5% defibered sheep blood and various concentrations of clarithromycin. Concentrations of clarithromycin were tested by doubling the dilutions from 0.016 to 0.5 mg/L. The plate was incubated at 37 °C under microaerophilic conditions (10% O_2_, 5% CO_2_, and 85% N_2_) for 72 h. The MIC was defined as the lowest drug concentration that prevented the visible growth of *H. pylori* isolates. *H. pylori* ACTC43504 was used as the standard for each set of MIC measurements.

### MPC determination

The determination of MPC was performed according to a method reported by Zhang et al. [38]. Briefly, *H. pylori* were cultured in brain heart infusion (BHI) broth containing 5% fetal bovine serum and incubated for 72 h. Cultures were centrifuged at 5000×*g* for 10 min, suspended in PBS, and adjusted to a concentration of 1 × 10^11^ CFU/mL. Next, 100 μL of the bacterial suspension containing 1 × 10^10^ CFU of bacteria was added to each MH agar plates containing 5% defibered sheep blood and various concentrations of clarithromycin. Concentrations of clarithromycin were tested by doubling the dilutions from 1 to 64-times the MIC. The plate was incubated at 37 °C under microaerophilic conditions (10% O_2_, 5% CO_2_, and 85% N_2_) for 72 h. The MPC was defined as the lowest concentration of drug that prevented the visible growth of *H. pylori* isolates.

### Statistical analysis

The univariate analysis was performed by using Kruskal-Wallis H Test. The analysis of the relation between MICs and MPCs was performed using Spearman rank correlation coefficient. A *P*-value of ≤0.05 was considered significant.

## Data Availability

All data generated or analyzed during this study are included within the article.

## Abbreviations

MIC: Minimum inhibitory concentration
MPC: Mutant prevention concentration
MSW: Mutant selection window
PCR: Polymerase chain reaction
PKPD: Pharmacokinetic and pharmacodynamics
